# The “angioletti” of Palermo: the health and development of mummified non-adults in late modern Palermo, Sicily (1787–1880 CE)

**DOI:** 10.3389/fmed.2024.1443291

**Published:** 2024-09-11

**Authors:** Kirsty Squires, Mark Viner, Wayne Hoban, Robert Loynes, Katherine Van Schaik, Dario Piombino-Mascali

**Affiliations:** ^1^School of Health, Education, Policing and Sciences, Staffordshire University, Stoke-on-Trent, United Kingdom; ^2^Cranfield Forensic Institute, Cranfield University, Cranfield, United Kingdom; ^3^Reveal Imaging Ltd., Whitley Bay, United Kingdom; ^4^KNH Centre for Biomedical Egyptology, The University of Manchester, Manchester, United Kingdom; ^5^Department of Radiology and Radiologic Sciences, Vanderbilt University Medical Center, Nashville, TN, United States; ^6^Department of Classical and Mediterranean Studies, Vanderbilt University, Nashville, TN, United States; ^7^Department of Electrical and Computer Engineering, Vanderbilt University, Nashville, TN, United States; ^8^Department of Anatomy, Histology, and Anthropology, Vilnius University, Vilnius, Lithuania

**Keywords:** mummies, non-adults, Capuchin Catacombs, radiography, demography, health, paleoradiography

## Abstract

The Capuchin Catacombs in Palermo, Sicily, have been home to non-adult mummified remains since the seventeenth century CE. Despite the increasing numbers of scientific studies conducted at this site, very little research has focused specifically on the youngest members of late modern (1787–1880 CE) society. This research aims to redress the balance by examining 43 individuals to gain insight into the demographic profile of mummified non-adults, to characterize their health status and possible cause of death, and to better understand the funerary treatment offered to the youngest members of society. A portable X-ray unit was used to capture anteroposterior and lateral images of each mummy; this facilitated age estimation, the identification of pathological and/traumatic lesions, and evidence of conservation and the mummification process more generally. This study revealed that regardless of age and health status at the time of death, the mortuary rite performed was primarily influenced by the wealth and social standing of the deceased’s kin. No demographic trends were observed in the data and the lack of evidence of metabolic, neoplastic, and traumatic bone lesions suggest these non-adults died from short-term, acute illnesses. Even when individuals did display evidence of chronic health conditions that would have impacted their day-to-day lives (e.g., B035), they were not excluded from this mortuary tradition on the basis of their long-term health and care requirements in life. Artifacts were found with all individuals examined and were associated with the mummification process, conservation of mummies, and/or their display. This research has ultimately demonstrated that non-invasive imaging can be used to gain a more comprehensive understanding of the lives and deaths of non-adults inhabiting late modern Palermo.

## 1 Introduction

Mummification has a long history in Christianity, with its practitioners citing a traditional Jewish custom of anointing the bodies of the dead, which may have resulted in at least partial retention of the soft tissues (1). However, it is the wish to venerate early martyrs’ relics that evolves into both spontaneous and anthropogenic preservation. This post-mortem phenomenon was the result of either the environmental conditions of the final resting place of prominent religious figures, or due to certain embalming practices conceived to spare cadavers from decomposition for worship and commemoration (2). Either way, bodies were perceived as sacred or holy and became the center of special attention, worthy of divine recognition (3). As a sophisticated funerary tradition, mummification was practiced in Palermo, Sicily, from the late sixteenth century to the early twentieth century. This practice was initially observed by the Capuchin Friars who exhumed a burial containing 45 well-preserved bodies. The outstanding preservation of these individuals was viewed as a sign from God, leading the friars to reinter the mummified remains behind the main altar at the Church of Santa Maria della Pace (4). This event consequently prompted the Capuchin Friars to mummify the dead. *Colatoi* (preparation rooms) were subsequently opened and used to prepare bodies of deceased friars. Spontaneous (or natural) mummification was primarily practiced and occurred from the natural desiccation of cadavers. In the case of the Catacombs, deceased individuals were placed in a preparation (or draining) room until they achieved an advanced stage of desiccation; they were then taken outside and washed with vinegar before being dressed (5). In some cases, this was followed by packing the bodies with straw and tow (a coarse fiber often used for stuffing) to maintain their shape. Anthropogenic (also termed artificial) mummification involved evisceration and/or chemical preservation. Initially, the mortuary rite of mummification was reserved solely for the clergy, though it was later offered to the nobility and the middle class as it was increasingly viewed as a status symbol and related to the worship of the “souls of the purgatory” (6). It was believed that by preserving the deceased, their social identity persisted after death and their souls became protective entities (7). As a result of these ideological beliefs and the social importance of mummification in late modern (1787–1880 CE) society, the dead were displayed in the Capuchin Catacombs where their relatives could visit them after death. Males, females, and non-adults were all mummified and dressed in their finery before being displayed at this site. At least 1,284 mummified and skeletonized bodies continue to be displayed in the Catacombs and, consequently, they form one of the largest and most important collections of mummies in the world owing to the assemblage size, unique nature of the mortuary rite, and insight it offers into late modern society in Palermo.

Extensive research has been conducted on the adults in the Catacombs, but the non-adults^1^ (*n* = 163) have largely been overlooked except in a small number of studies [e.g., (8–12)]. Indeed, the relative lack of interest in and study of pediatric remains has been a common theme within mummy studies more broadly (13), although such oversight is now being rectified [e.g., (14–24)]. This lack of knowledge of the non-adults in the Capuchin Catacombs is compounded by the limited documentation for non-adults who were granted mummification. Death records from the period of interest contain only the name and date of death of the deceased, with very little information about funerary rites (25). Moreover, broader knowledge of non-adults living and dying in late modern Palermo is limited by the number of studies that focus on this demographic group [e.g., (26–30)]. Research to date has focused primarily on individuals of a lower social standing, in contrast to their middle- and upper-class counterparts. Analysis of non-adult remains is therefore necessary to understand their bio-histories and relative social standing. In the present study, a portable direct digital X-ray device was used to obtain radiographs of 43 non-adult mummified bodies^2^ with the aim of shedding light on the demographic attributes of these individuals, their health status at the time of death, and, where possible, their cause of death.

## 2 Materials and methods

In total, 43 non-adults were examined in this study. Individuals in the ‘Children’s Room’ were selected, as well as one non-adult (B001) from the ‘Men’s Corridor,’ because of their accessibility. Individuals selected for analysis from the Children’s Room were displayed in niches or open coffins. At the time of analysis, the Children’s Room contained several (*n* = 7) closed coffins, the contents of which are unknown because these were not opened, meaning the number of individuals in these caskets, and their preservation status, cannot be commented upon. All images and provisional anthropological data were obtained *in-situ* in the Capuchin Catacombs as it was not possible to remove the mummies from this context because of ethical considerations, ideological beliefs, and health and safety concerns (7). Prior to radiographic evaluation, individuals were triaged based on the potential complexities of obtaining images of the mummified remains. Each mummy was assigned a ‘Bambino’ (B) number, and a triage form was completed. The project’s radiographers (MV and WH) established the required radiographic views for each individual and assisted in mitigation of the effects that acquisitional artifacts (from positioning, overlapping structures, and non-skeletal material) might have on the images (34). During this process, individuals were assigned a triage level based on the intricacy of each case (e.g., completeness, state of preservation, position of the body). The implementation of this system in the initial stages of the investigation improved overall efficiency and organization. Each mummy was photographed *in-situ* and initial bioarchaeological with a assessment was completed, focusing on visible dental eruption and development (35).

A radiation-controlled area was cordoned off and access-controlled for the duration of analysis. A portable X-ray unit was set up in the ‘Professionals’ Corridor as there was access to power and no mummies in the niches of the selected area. The X-ray unit (an Xograph DRgo system) consisted of a Canon Lanmix CDXi 35×43 cm direct digital radiography plate and Canon CDXi acquisition software. The X-ray tube used was a Sedecal SP4 4 kW stationary anode high frequency generator. DICOM files were viewed and exported using Merge Efilm software. Anteroposterior (AP) and lateral (from the head to toe) images were acquired of each mummy in order to estimate age and sex and to identify pathological and/or traumatic bone lesions. These images focused upon the epiphyses of the major long bones (e.g., femora, humeri), carpals, vertebrae, and pelves, as these are most appropriate for estimating the age of younger individuals. In addition, oblique mandible and intra-oral dental radiographs were generated where possible for the purpose of age estimation using dental eruption and development criteria. When soft tissue survived in the pelvic area, it was thought possible to determine the sex of the child through radiographic examination of external genitalia. The dental X-ray system consisted of a SIRONA photostimulable phosphor plates (PSP) system, scanner, and acquisition software. These radiographs were generated using the same Sedecal X-ray tube detailed above. Radiographs also targeted areas of interest, e.g., crania, to further understand the presence of pathological and traumatic lesions.

Radiographs were examined using a DICOM reader (Osirix MD v 13.0.3) by a multi-disciplinary team of medical professionals and bioarchaeologists. This interdisciplinary, consensus-based approach is acknowledged to be an essential best-practice method, given atypical imaging acquisition planes, post-mortem decay, and presence of artifacts associated with the mummies for conservation and display purposes (36). Radiographs were used for age estimation (35, 37–39), sex determination (where soft tissue survived), and the identification of pathology, trauma, and personal effects (including mortuary artifacts). It must be stressed that there are limitations to each of the age estimation methods used; hence a combination of techniques was adopted in this analysis to improve the accuracy of the resultant age estimates (40). Interpretation of pathology was carried out in the context of the Index of Care as developed by Tilley and Cameron (41).

## 3 Results

### 3.1 Demography

Age estimation of the 43 non-adults showed individuals ranged from newborn to nine years old (Table 1). However, the majority of non-adults examined in this research were under five years of age (*n* = 32; 74.4%^3^). In some cases, the age assigned to an individual was broad (e.g., B007; B021) due to the incomplete nature of the mummy, lack of dentition, and/or absence of dental radiographs (i.e., if it was not possible to access the oral cavity). There was no association between the placement of individuals in the Children’s Room and age. Biological sex was ascertained through an examination of soft tissue where possible. This was only feasible in a small number of cases (*n* = 4, 9.3%), all of whom were identified as male (B001; B029; B042; B043). Clothing could not be used to accurately determine sex or gender, as individuals only started wearing gender-related clothing from five years of age and, as we observed, the majority of individuals in the Children’s Room would fall into this more gender-neutral grouping (42). In a small number of instances, clothing was suggestive of gender (female: B013; B027; B028; B032; B033 or male: B029), though these observations have not been included in the final conclusions as these are only tentative findings. Consequently, further interpretation of the sex or gender of non-adults was not possible.

**Table 1:** Results of anthropological and radiological analyses of the non-adult mummies from the Capuchin Catacombs.

Mummy No.	Age	Sex	Pathology	Internal organs	Mummification type	Categories of artifacts
B001	6 months–18 months	Male	None	Brain; lungs	Possible anthropogenic	Metal wire
B002	18 months–2 years	Unknown	None	No	Spontaneous	Metal wire, stake
B003	2 years–4½ years	Unknown	None	No	Spontaneous	Metal wire
B004	3 years–5 years	Unknown	Harris Lines	No	Spontaneous	Metal wire, wooden board
B005	2 years–3 years	Unknown	None	No	Spontaneous	Metal wire, stake, cane, nail
B006	3 years–4 years	Unknown	None	No	Spontaneous	Metal wire, stake, cane, nail/peg
B007	1 year–6 years	Unknown	None	No	Spontaneous	Metal wire, stake, nail
B008	7½ months–2 years	Unknown	None	No	Spontaneous	Metal wire
B009	2½ years–3½ years	Unknown	None	No	Spontaneous	Metal wire, stake, cane, nail, wooden board
B010	2 years–4½ years	Unknown	None	No	Spontaneous	Metal wire
B011	4 years–5 years	Unknown	None	No	Spontaneous	Metal wire, nail, stake, wooden board, packing
B012	2 years–4½ years	Unknown	None	No	Spontaneous	Metal wire, stake, cane, metal fastener, packing, fine wire
B013	6 years–8 years	Unknown	Harris Lines	No	Spontaneous	Metal wire, stake, cane, metal fastener, nail, wooden board, packing, fine wire
B014	1½ years–3 years	Unknown	Harris Lines	No	Spontaneous	Metal wire, cane, stake, nail, wooden board, packing, fine wire
B015	3 years–5 years	Unknown	None	No	Spontaneous	Metal wire, stake, metal fastener, packing
B016	2 years–6 years	Unknown	None	No	Spontaneous	Metal wire, cane, wooden board, nail, metal fastener, pin, packing
B017	2 years–3 years	Unknown	None	No	Spontaneous	Metal wire, stake, metal pin, wooden board, nail
B018	7 years–9 years	Unknown	None	No	Spontaneous	Metal wire, nail, wooden board, packing
B019	2½ year–5 years	Unknown	None	No	Anthropogenic	Metal wire, metal fastener
B020	3 years–5 years	Unknown	None	No	Spontaneous	Metal wire, nail, fabric thread (to tie sleeve cuffs)
B021	4 years–7 years	Unknown	None	No	Spontaneous	Metal wire, wooden board, nail, stake, packing, fine wire
B022	2½ years–4 years	Unknown	None	No	Spontaneous	Metal wire, pin, stake, packing
B023	3 years–5 years	Unknown	Harris Lines	No	Spontaneous	Metal wire, stake, packing
B024	2 years–4½ years	Unknown	None	No	Spontaneous	Metal wire, cane, wooden board, metal fastener, packing, fine wire
B025	2 years–3½ years	Unknown	Periosteal reaction on left proximal humerus, possibly indicative of osteomyelitis, Ewing’s tumor, eosinophilic granuloma or osteosarcoma	No	Spontaneous	Metal wire, cane, stake, nail, packing, fine wire
B026	3 years–4½ years	Unknown	None	No	Spontaneous	Metal wire, cane, stake, nail, packing, fine wire
B027	4½ years–6½ years	Unknown	None	No	Spontaneous	Metal wire, stake, wooden board, metal fastener, packing, fine wire
B028	4½ years–6 years	Unknown	None	No	Spontaneous	Metal wire, stake, nail, metal fastener, wooden board, packing
B029	7 years–8 years	Male	Scoliosis	No	Anthropogenic	Metal wire, wooden board, nail
B030	4 years–5 years	Unknown	None	No	Spontaneous	Chair
B031	6 months–12 months	Unknown	None	No	Anthropogenic	Chair (same chair as B030)
B032	7 years–9 years	Unknown	None	No	Spontaneous	Wire, stake, packing, fine wire
B033	5 years–8 years	Unknown	None	No	Spontaneous	Wire, cane, stake, nail, metal fastener, pin, packing, fine wire
B034	2 years–4 years	Unknown	Harris Lines	No	Anthropogenic	Wire, metal fastener
B035	2½ years–4½ years	Unknown	Dramatic pes cavus deformities; Harris Lines	No	Anthropogenic	Wire
B036	2 years–3 years	Unknown	None	No	Spontaneous	Wire, nail, cane, stake, packing, fine wire
B037	3 years–4½ years	Unknown	None	No	Spontaneous	Wire, nail, cane, stake, packing, fine wire
B038	3 years–5 years	Unknown	None	No	Spontaneous	Wire, stake, cane, nail, packing, fine wire
B039	2 years–4 years	Unknown	None	No	Spontaneous	Wire, nail, stake, wooden board, packing
B040	2 years–4 years	Unknown	None	No	Spontaneous	Wire, stake, nail, wooden board
B041	4 years–6 years	Unknown	None	Brain; organs in thoracic and abdominal cavities	Anthropogenic	Coffin, blanket
B042	Neonate–4 months	Male	None	Brain	Anthropogenic	Crib, blanket, pillow, dense material (unidentifiable) in right fifth pedal digit
B043	Neonate–3 months	Male	None	No	Anthropogenic	Crib, blanket, pillow

### 3.2 Health and disease

**Figure 1:**
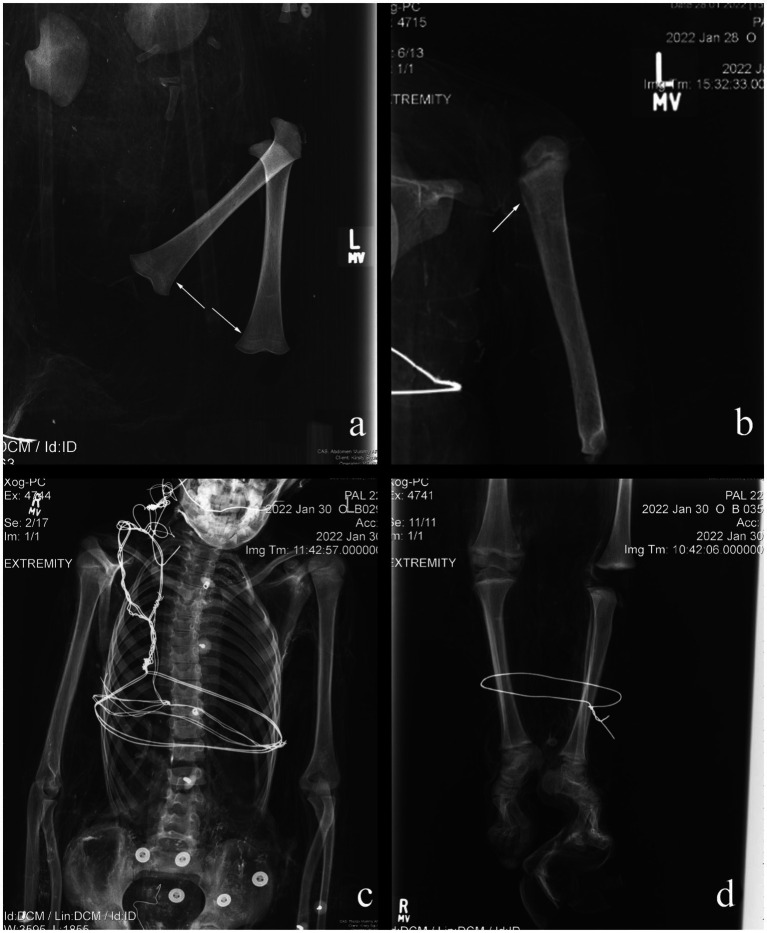
Examples of pathological conditions found in the investigated non-adult mummies. **(A)** B014: Harris Lines; **(B)** B025: periosteal reaction; **(C)** B029: scoliosis; **(D)** B035: pes cavus.

Overall, the individuals examined in this study exhibited little radiographic skeletal pathology and trauma. In total, six non-adults (14.0%) exhibited Harris Lines. Harris Lines were observed in individuals ranging from one and a half years of age to eight years and are most frequently seen in three- to four-year-olds (Figure 1A). Three non-adults showed evidence of more unique pathological conditions. Non-adult B025 presented periosteal reaction on the left proximal humerus, for which the differential diagnosis includes osteomyelitis, Ewing’s tumor, or eosinophilic granuloma, given the location of the lesion and the individual’s age (Figure 1B). Each of these potential diagnoses would have caused pain and therefore, could have led to reduced function and movement. Minor scoliosis was observed in individual B029 (Figure 1C). This was identified by the ‘S’ shaped curve to the spine and was almost certainly asymptomatic. Given the minor nature of the scoliotic curve, it is unlikely that the individual would have been in pain or exhibited an apparent physical deformity. It is thus plausible that the individual could have easily participated in daily activities. Non-adult B035 demonstrated Harris Lines on the proximal and distal tibiae alongside bilateral marked pes cavus deformities, suggestive of a neuromuscular abnormality, including but not limited to polio, Charcot–Marie-Tooth, or cerebral palsy (Figure 1D). The individual would likely have experienced pain with ambulation, not only because of the underlying neuromuscular condition, but also because of the altered mechanics associated with the pes cavus deformity. Individuals with these types of disorders often display an abnormal gait.

### 3.3 The mummification rite and funerary traditions

**Figure 2:**
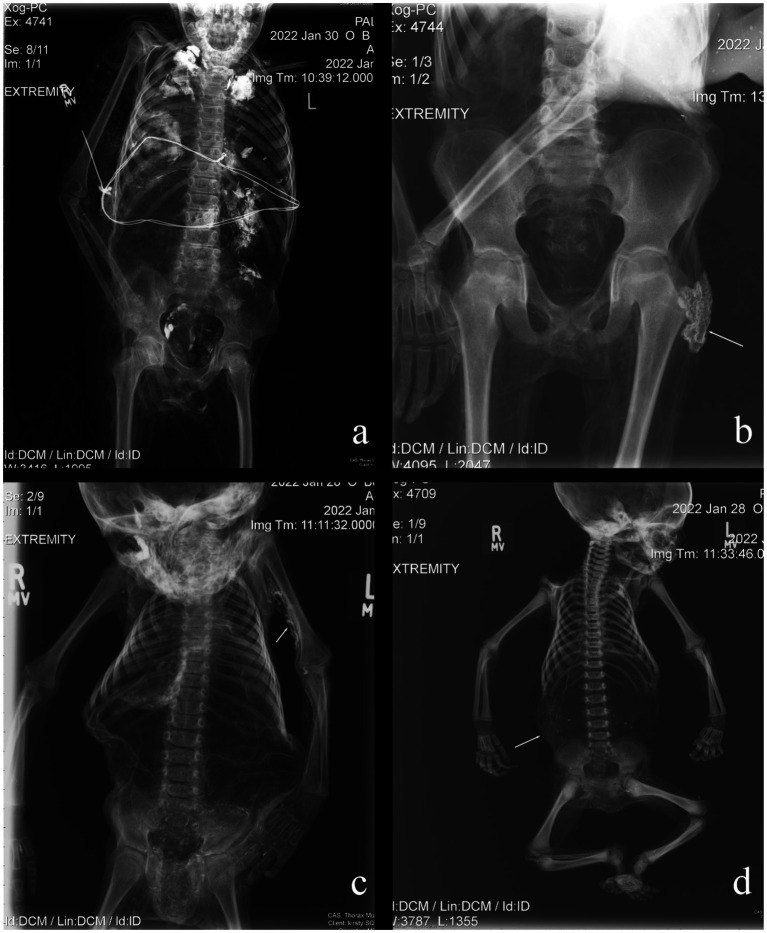
Examples of radio-opaque material identified on the non-adult mummies examined in this study. This may derive from preservatives, such as hydrated lime, which has dried onto the body’s surface following immersion. **(A)** B035: trunk; **(B)** B041: pelvic region; **(C)** B042: upper arm; **(D)** B043: abdomen.

**Figure 3:**
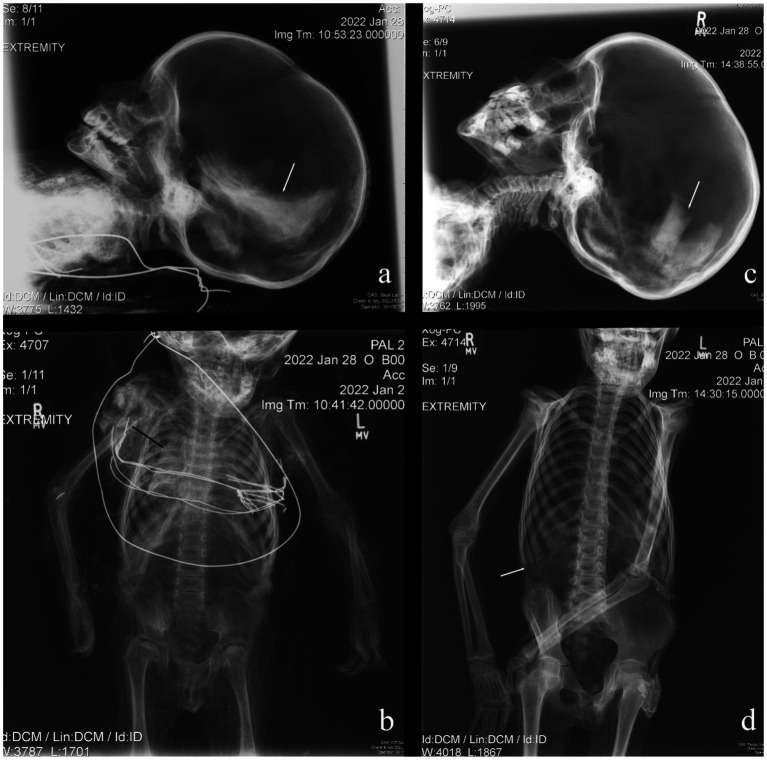
Evidence of organ preservation in two non-adults. **(A)** B001: brain; **(B)** B001: lungs; **(C)** B041: brain; **(D)** B041: thorax and abdominal cavities.

Both spontaneous and anthropogenic mummification were used in the mortuary rites of the non-adults in the Capuchin Catacombs. In total, eight (18.6%) individuals showed some macroscopic evidence of anthropogenic mummification by the presence of an incision on the neck, by the texture and color of the skin, as well as remarkable preservation of their features. Sometimes the evidence collected was less obvious. Non-adult B001 was possibly offered this mortuary treatment, though this was difficult to ascertain due to the dress the individual was wearing. Different individuals, such as B035 and B041, were likely to have been anthropogenically mummified. This is suggested by a type of high-density material observed in several areas (Figures 2A,B), though in some cases this may have consisted of an external treatment, such as dipping the bodies into preservatives (e.g., hydrated lime) (Figures 2C,D). Specifically, radio-dense material was identified on and/or near the body’s surface of non-adults B042 (medial surface of the left humerus and anterior pelves) and B043 (abdomen). Of the nine assumed anthropogenic mummifications, four (44.4%) showed evidence of this material. Spontaneous mummification was used for the remaining individuals (*n* = 34; 79.1%), all of whom are in variable states of completeness and preservation, with some partially skeletonized. This is in stark contrast to the anthropogenic mummies, which are largely complete and well-preserved. Internal organs were only identified in two (4.7%) individuals: B001 contained a preserved brain and lungs (Figures 3A,B), while the brain and organs in the thoracic and abdominal cavities of B041 were recorded (Figures 3C,D). Interestingly the latter individual was anthropogenically mummified and as previously mentioned, it is possible that the same funerary treatment was used for B001. However, there is no association between mummification type or state of preservation and age of the deceased.

**Figure 4:**
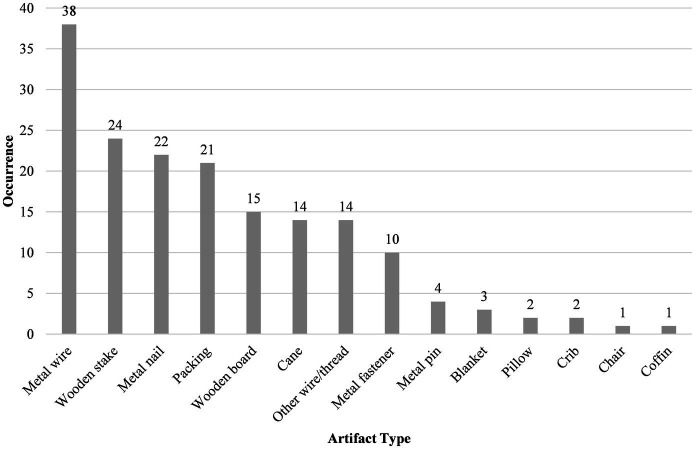
Types of artifacts associated with non-adult mummies in the Capuchin Catacombs. Each class of object has been counted once per individual; multiple occurrences of each artifact type from a single individual have been excluded.

**Figure 5:**
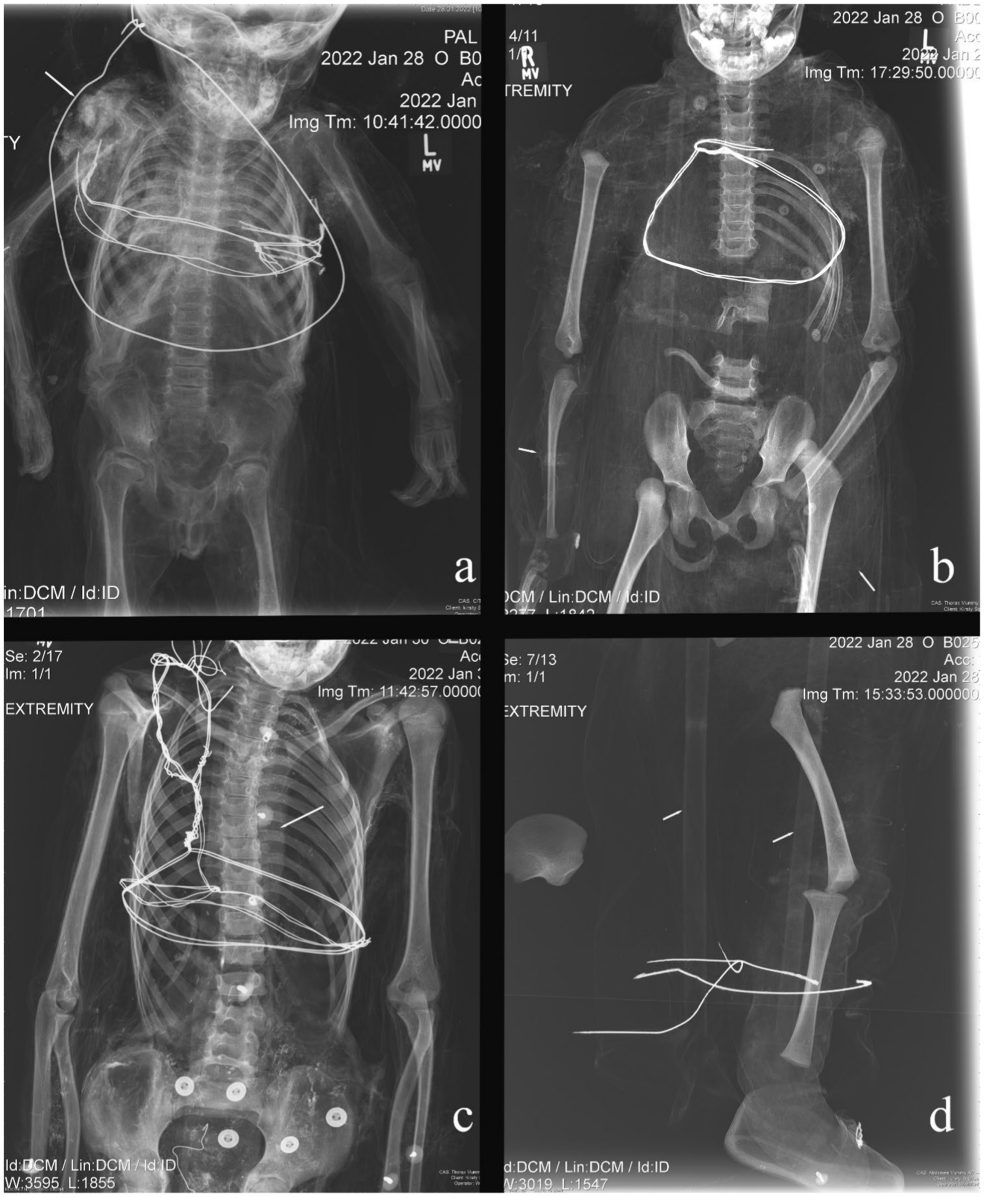
Materials (artifacts) used following mummification. **(A)** B001: suspension wire; **(B)** B003: fine wire used to shape appendages; **(C)** B029: wooden board; **(D)** B025: canes.

Artifacts were frequently recorded with the non-adult mummies (Figure 4). These objects had one of two key functions, either as a means of conserving the mummified remains or to display the deceased. Anthropogenic mummies are associated with fewer artifacts, such as wire and metal fasteners, than spontaneous mummies as they tend to be better preserved and conservation attempts have not been made. Instead, they are more likely to be associated with atypical forms of display, such as placement in open coffins and cribs with blankets and pillows. In contrast, spontaneous mummies (*n* = 35; 81.4% of the studied assemblage), and a small number of anthropogenic mummies (B019 and B035), were displayed and hung in niches in the Children’s Room using metal wire (Figure 5A). These individuals were frequently found with wire (Figure 5B), wooden stakes, metal nails, tow, wooden board (Figure 5C), and canes (Figure 5D). There was no clear relationship between age and use of different categories of artifacts. However, only the youngest individuals were placed in a coffin (B041) or crib (B042; B43), while B031 sat on the lap of B030 who was positioned on a chair. Associations between sex and artifacts have not been attempted because of the small number of individuals assigned sex in this study.

## 4 Discussion

In this research, three distinct, yet interrelated themes have emerged and provide an unprecedented understanding of middle- and upper-class children in late modern Palermo.

### 4.1 Demography

The Capuchin Catacombs are home to a total of 163 non-adult mummies, with at least 42 of these individuals in the Children’s Room at the time this study was carried out. It would appear that the selection of individuals for display in this area was less likely to have been influenced by their age, than by their height and ability to be positioned upright in the niches in this room. Death records from 1819–1856 CE, which are held in the library of the Capuchin Convent in Palermo, were examined to establish an approximate proportion of mummified non-adults. These tomes detail the deceased’s name, year of death and, on occasion, instances of anthropogenic mummification, and the draining room used. In total, approximately 9,300 non-adults were buried in the Catacombs from 1819–1856 CE. Here, it is worth highlighting that during this time frame (1826–1849 CE), a cholera epidemic had badly affected Palermo (and Sicily more broadly), resulting in the death of over 24,000 individuals (43). Thus, the number of non-adults counted from the death records may be higher than expected due to this outbreak. Nonetheless, if we consider the number of non-adults recorded and the number of young individuals in the Catacombs, approximately 1.7% of a subsection of the non-adult population of late modern Palermo is currently represented in the crypt, while the remaining 98.3% would have been inhumed in family plots or mass graves, or were lost over time (44). This low figure is unlikely to be the result of the living not mummifying young individuals on the basis of their age. Indeed, in the late modern period it was believed that deceased non-adults acted as a mediating force between the living and the afterlife and were viewed as ‘little angels’ (angioletti) (31). Instead, their social class and family’s wealth was likely to have been more influential in the choice of funerary treatment than their biological age.

### 4.2 Health and disease

One of the most striking findings of this study has been the dearth of radiographically apparent skeletal pathology. This indicates that most non-adults would have died from short-term, acute conditions that typically do not leave skeletal evidence, for example, cholera and smallpox. Indeed, infectious diseases, such as tuberculosis, smallpox, and scarlet fever, were the primary cause of non-adult (0–14 years) mortality in Italy between 1887 and 1889, amounting to 20% of all deaths during this period (45). These types of diseases can cause a rapid decline in health prior to death and, as such, skeletal lesions do not have time to develop as a result of these conditions.

As mentioned above, a cholera epidemic gripped Sicily in the first half of the nineteenth century, with subsequent outbreaks in the latter half of the century (46). Cholera is caused by the bacterium *Vibrio cholerae* and is spread through contaminated food and water (47). The most common symptoms experienced by infected persons are severe diarrhea and vomiting over a very rapid time frame; therefore, the disease leaves no pathological markers on the skeleton (48). While a number of measures were introduced to prevent the spread of the disease, such as quarantines and disinfection measures, these plans were rarely effective (43, 49). Poor and marginalized individuals were primarily affected by cholera, although the middle-classes and nobility were not immune from contracting the disease [e.g., (43)]. Given the frequency of these epidemics and the absence of skeletal lesions associated with cholera, we cannot rule out that some of the non-adults included in this research died from this disease. Smallpox is an airborne disease caused by the variola virus, and can lead to skin lesions, blindness, limb deformities, ankylosis, and death (50). Non-adults were at a higher risk of contracting and transmitting smallpox than adults as they were less likely to have acquired immunity from earlier exposures. As a result, a vaccination campaign, primarily targeting the young, was launched in Palermo in 1801 CE (51). The vaccination status of the non-adults examined in this study is unknown, though the relative absence of radiographic lesions (e.g., osteomyelitis) suggestive of smallpox may indicate they were vaccinated. Furthermore, inoculations against the disease became compulsory in 1821 CE (52, 53), which would have protected individuals from falling ill and subsequently developing the skeletal lesions that can be associated with the condition (54). Still, there is also the possibility that individuals were unvaccinated (e.g., those that lived before 1821 CE or had recently moved to Sicily) and died from the disease before any bony changes could take place (48, 55).

There is a relative lack of skeletal evidence of nutritional deficiency in the images acquired in this study, which perhaps is not surprising, given the children’s social status (8). During the late modern period, the middle-classes and nobility would have had access to a range of foodstuffs, including pasta, bread, vegetables, fruit, legumes, game, and fish (56). In contrast, those not as fortunate as the upper echelons of Palermo society were either consuming a nutritious, albeit more restricted, diet (as was the case for female orphans working in the silk industry) or were living hand to mouth (30, 57). Six individuals from the study sample did exhibit Harris Lines, which may indicate continued or catch-up growth after temporary growth arrest, possibly in the setting of nutritional deficiency or periods of physiological and/or emotional stress (such as illness). Mary Lewis (55) has noted that Harris Lines formed between five to nine years of age are likely to be the result of a pathological event as opposed to a growth spurt, which is seen in adolescence. Such an interpretation could potentially apply to B013 (6 years–8 years), B004 (3 years–5 years), and B023 (3 years–5 years), if their ages are at the older end of the estimates noted in this study. In the absence of pathological skeletal markers in these individuals, with the exception of B035 (pes cavus deformities), it is not possible to confidently state why these non-adults developed Harris Lines. If these were pathological in nature, individuals would have needed an adequate diet, rest, and a clean environment if they were to recover (41). These potentially stressful episodes may have led to immune-related physiological changes in the body which led to a reduced immune response and, ultimately, premature death (58).

Three non-adults (B025, B029, and B035) exhibited radiographically-apparent pathological findings. B025 demonstrated periosteal reaction in the proximal humerus, which may have been accompanied by pain and swelling in the upper limb. This, in turn, could have led to “protection” of the limb and subsequent disuse. Depending on the etiology of the periosteal reaction, other symptoms could have included fever. If eosinophilic granuloma were the cause, then spontaneous resolution may well have occurred (59). However, were the cause to be osteosarcoma, then early death would have resulted. Osteomyelitis, if accompanied by sepsis, would also have led to death. Yet, if the infection remained local, then chronic osteomyelitis would have ensued, possibly with a suppurating wound. Treatment of osteosarcoma and osteomyelitis in nineteenth-century Sicily would have been limited. The Academy of Studies in Palermo taught medicine and surgery, meaning doctors from this institution would have been able to attend wealthy families in the city (60). Herbal preparations, rituals, prayers, and bloodletting may have also been used in an attempt to heal non-adults suffering from neoplastic and infectious conditions (61, 62).

B029 showed signs of very mild juvenile scoliosis (63) which would almost certainly not have been noticed by parents even when the child was unclothed. Likewise, the child was probably unaware of this condition as it would have been entirely asymptomatic (64), without inhibition of daily activities or the need for spine-straightening therapy [e.g., (65–67)]. This is in stark contrast to B035, in whom bilateral pes cavus was identified. Such findings could have resulted from a neurological condition that would have led to muscle spasm or muscle imbalance (68). Some of the potential causes of this condition are mentioned above. Pain is an unfortunate feature of pes cavus, a consequence of the underlying nerve disorders, muscle contractures, and biomechanical alterations that compromise gait (69). If this individual did suffer from a neuromuscular disorder, then attendant muscle weakness and/or spasticity in the lower limbs would have contributed to an ataxic gait that may have affected other joints more proximally (70). Poultices may have been applied to the feet in the same way they were employed for sprains (62). In the late modern period, a range of treatment methods were available and primarily involved stretching the feet manually or on rack-splints, manual wrenching, and use of supports to lift the anterior arch (71). However, these methods were rarely long-lasting. Consequently, the more likely outcome may have been use of a wheelchair or sedan chair (72, 73). There was still some stigma associated with disabled individuals among the poor in the nineteenth century (74), though the high social standing of the individual in question may have relieved them from some dimensions of such prejudices.

Even if they had longstanding health issues, non-adults were not excluded from burial in the Capuchin Catacombs. Similarly, age does not appear to have excluded an individual from the rite of mummification in late modern Palermo. This highlights two important points. Firstly, while we know deceased non-adults were revered, the decision to mummify individuals was heavily based on the social status of the deceased and/or their family regardless of demographic traits or health status during life. Secondly, instead of being ostracized or treated differently, non-adults who had chronic conditions and physical differences (e.g., unique gait, morphological differences, and possible soft tissue lesions that may or may not have contributed to their death), were given the same funerary rite as non-disabled individuals. This has also been observed by Panzer et al. (9) who examined a child exhibiting Robinow syndrome from the Capuchin Catacombs. Given the elaborate mortuary treatment of these non-adults, it is likely that they received appropriate care and medical treatment from their families and physicians. In stark contrast, children belonging to lower-class families would not have received the same level of care as adults; children from lower social strata likely had to work in physically demanding jobs, and their families were unlikely to have been able to afford treatments or doctor visits; their options were thus more limited to traditional therapeutic methods (62, 75).

### 4.3 The mummification rite and funerary traditions

No clear associations between demographic attributes of the deceased and the type of mummification used on non-adults in the Capuchin Catacombs were identified in this research. Anthropogenic mummification was mostly administered in the form of injection or immersion into preservatives such as arsenic, mercury, or hydrated lime (9, 44, 76). It is worth highlighting here that there were no radiological signs of mercury in the non-adults examined in this study. However, the radio-dense material observed in several individuals could potentially be embalming residues (9). In the majority of these cases, anthropogenic mummification was a more complex and expensive procedure than spontaneous mummification (77). It is therefore possible that anthropogenic mummification was selected to show social status and wealth of an individual and their family [e.g., (15, 53)]. This form of bodily preservation would have been more effective at preserving the soft tissues and would have turned the remains into those of a sort of familial saint, possibly reinvigorating political power and prestige among Palermo’s stratified society (31). Lastly, an emotional response toward grief may have also been a motivator, in that family members may have wished to preserve their young children as well as possible, in order continue to see the child’s physical likeness after death (78).

While this research focused on non-adults primarily from the Children’s Room, it is important to highlight that it is possible these individuals were originally placed elsewhere. This is supported by visitor accounts at the time. Nathaniel Parker Willis, a writer from the United States of America, offered an account of the Catacombs from his travels around the Mediterranean in 1853, and recounted the presence of non-adults in the Catacombs:

“*Upon a long shelf above sat perhaps a hundred children from one year to five, in little chairs worn with their use while in life, dressed in the gayest manner, with fanciful caps upon their little blackened heads, dolls in their hands, and in one or two instances, a stuffed dog or parrot lying in their laps.”* [(79), p. 50].

In the present day, the aforementioned shelf containing dozens of non-adults is no longer in use and these individuals may have been moved elsewhere and repositioned throughout the Catacombs. Indeed, mummies have also been moved as recently as the past two decades, as exhibited from more recent photographs of the Children’s Room [e.g., see (12)]. It is of note that, based on Willis’ description, many children were once positioned on chairs, though presently, very few are positioned this way. In the Children’s Room, only B030 (and B031) were seated on a chair, and elsewhere there are no seated children. The reasons for these changes can be ascribed to preservation of the remains; mummies with more advanced post-mortem decay were subsequently displayed elsewhere in the Catacombs or removed and buried in mass graves (80). Compromised preservation and the incomplete nature of some of the non-adults examined in this study can also be attributed to early visitor behavior, in which mummies were picked up and shown to guests, some of whom removed parts of the mummies as mementos of their visit (7, 80). This is illustrated when artist and topographer Cooper Willyams visited the Capuchin Catacombs in 1798, writing:

*“Our conductor took from a small coffin the remains of a young prince dressed in the fashion of his day, and presented it to the ladies as a toyman in London would have shown a doll. Unfortunately the young gentleman, perhaps from too rough treatment at other times, dropped his head, which fell forwards, to the no small alarm of his fair visitors.”* [(81), p. 177].

The items associated with the non-adult mummies were likely to have been used shortly after mummification to ensure the bodies could be hung on the walls in an upright position. It is also possible that some of them were added at a later stage if the mummies got relocated. Some of the artifacts found with non-adults can be linked to conservation attempts. The relatively poor preservation of desiccated individuals necessitated these items to improve the integrity of remains and to give the mummies a ‘fuller’ appearance; for example, stakes were placed in the neck, extending through the foramen magnum, so the skull would not fall away from the post-cranial body, a problem described in Willyams’ (81) account. Canes, tow, and straw were utilized to maintain the form of the body, while wire was employed to hold these materials together. In some cases, individuals were fastened to wooden boards using nails or wires to ensure the body did not fall apart. The use of these types of objects in the preparation process, and to support them as they are displayed, can be seen elsewhere [e.g., (82, 83)]. There are two main reasons to conserve these non-adult mummies; firstly, to uphold an agreement with kin who paid for the mummification, display, and care of their relative (12), and secondly, to ensure that the presence of visible mummified remains will continue to draw income from visitors, which funds upkeep of the structure and the mummified remains, and the friars’ charity work (7, 84).

Other artifacts associated with the non-adults examined in this study are more closely tied to the deceased’s stage in the lifecycle at the time of death. The dolls and stuffed animals mentioned in Parker Willis’s (79) account, for example, are no longer displayed, but these may have represented the non-adult’s favorite toy, or could have been a gift from mourners, a practice that dates to the Roman period and continues in the twenty-first century (85). Several of the youngest individuals were associated with either a crib (e.g., B042 and B043) or sat on the knee of an older child positioned on a chair (e.g., B031 sat on the lap of B030). The relationship between B030 and B031 is unknown. They may have belonged to different families with distinct customs, beliefs, and cultures (15), as B031 was anthropogenically mummified while B030 was spontaneously mummified. As such, these individuals may have died around the same time or the completion of mummification for both non-adults occurred concurrently, and therefore, they were displayed together. Alternatively, these young people may have once been seated on their own individual chairs but were subsequently manipulated for display purposes. There is evidence of manipulating and moving other young mummies. For example, B041 was once hung in a niche but was placed in a coffin several years ago. Where individuals were found in cribs (e.g., B042, B043) or coffins (e.g., B041), they were associated with blankets, while those in cribs were found with pillows. The placement of non-adults in an open crib or coffin with blankets strongly resonates with the ‘first style’ of nineteenth century death photography in which children appear to be sleeping (86, 87). Akin to the intentions of this kind of early nineteenth-century post-mortem photography, the placement of individuals on chairs, cribs, and open coffins may have served to comfort relatives in the face of such profound loss (88). It is worth highlighting that infants were, at times, hung in niches (e.g., B002, B008), thus the young age of some individuals did not exclude them from this form of display. It is possible that the Capuchin Friars placed individuals on chairs or in cribs when organizing and displaying mummies based on the availability of these items and space in the Catacombs.

## 5 Conclusion

Mummification has a long history in Sicily, and its use as a mortuary rite was reserved for those that could afford this form of funerary treatment, i.e., the clergy, middle-classes, and nobility. This research has demonstrated that mummification, and the display of mummies, was very much a minority rite, with less than 2.0% of non-adults buried in the Catacombs treated in this manner. The selection of anthropogenic or spontaneous mummification was not influenced by age of the deceased and appears to be more closely related to the social standing of the deceased’s family and what they wished to invest in the funeral. The non-adults examined in this research exhibited very little evidence of chronic metabolic diseases, which is to be expected given that they belonged to middle-class families or the nobility. There is evidence that non-adults with chronic health conditions were mummified, highlighting that they were not excluded from this kind of post-mortem treatment because of their health status. In each case, any potential cause of death which may have been reflected in the skeleton was not identified, which suggest these individuals died from acute conditions, such as cholera or smallpox. An examination of individuals from the Children’s Room revealed a variety of artifacts attributable to the mummification process, to the conservation of mummies, and to the display of non-adults in niches or in cribs, coffins, or on chairs. This research has ultimately highlighted the great role that wealth and familial status played in the decision to mummify non-adults: mummification practices do not appear to have been withheld from elite children because of age or of their health status before or after the time of death. Further investigations focusing on cause of death and associated artifacts for other non-adult mummies throughout the Capuchin Catacombs and elsewhere in Sicily would offer a more in-depth understanding of the lives and deaths of these young individuals during the late modern period.

## Data Availability

The original contributions presented in the study are included in the article/supplementary material, further inquiries can be directed to the corresponding author.

## Footnotes

^1^For the purpose of this paper, ‘non-adult’ refers to all individuals under the age of 18 years old.

^2^The most well-known non-adult from the Capuchin Catacombs, Rosalia Lombardo, was not examined in this study as this individual has been extensively studied elsewhere [e.g., see (9–11,31–33)].

^3^This figure only includes individuals where age was confidently identified as five years and younger; those assigned an age range that went beyond five years old (e.g., 4–6 years) were excluded from this estimate.
